# Supplementary material for: Multidisciplinary Intensive Rehabilitation Program for People with Parkinson’s Disease: Gaps between the Clinic and Real-World Mobility
Source: Int J Environ Res Public Health. 2023 Feb 21;20(5):3806. doi: 10.3390/ijerph20053806 (PMC10001519; doi:10.3390/ijerph20053806)
Supplement: Supplementary file 1 [file ijerph-20-03806-s001.zip › ijerph-2110978-supplementary.pdf]

Supplementary Material

METHODS

Daily-living Axivity Features Overview

The measures derived from the Axivity recordings can broadly be divided into two families of metrics:

1) Gait quality (the quality of the walking pattern)
2) Activity and gait quantity (e.g., total daily-living physical activity, step counts)

The gait quality measures are determined in each walking bout across the recording of a relatively long duration (e.g., 60 seconds) and then the mean, median, 90%, 10% and standard deviation of each metric across all walking bouts of that size are determined.

Gait quality measures can be further subdivided into several subdomains:

a) Pace: e.g., gait speed and step length
b) Rhythm, e.g., step time, stride time, cadence, dominant frequency
c) Symmetry, e.g., step regularity, step symmetry, harmonic ratio
d) Complexity, e.g., entropy

The gait and activity measures are described in further detail below. See Galperin et al. [1] for additional description of the process:
https://www.sciencedirect.com/science/article/pii/S1353802019300227?via%3Dihub

Gait Quality Measures

Rhythm Measurements

Cadence [steps/min]

In daily life experiments (data recorded from Axivity device), we determine cadence from the frequency domain. We identified the dominant frequency of the signal, and calculate the inverse value of the dominant frequency, to determine the average step time.

Mean stride time [sec]

Time of average gait cycle (e.g., heel-strike to heel-strike of the same foot).

Symmetry Measurements

Step symmetry [nu]

Expresses the symmetry of the acceleration between left and right limbs.

Step regularity [nu]

Expresses the regularity of the acceleration between consecutive steps. Low step regularity indicated that there is a low regularity between steps or a systematic asymmetry between left and right leg.

Complexity Measurements

Entropy and Sample Entropy [nu]

These measure the complexity of the signal. Entropy is a measure that quantifies regularity in time series: the more predictable and less complex a series is, the lower the entropy value. For a given dimension m, parameter that defines the criterion of similarity r and the number of samples N, sample entropy is negative logarithmic of the probability that if two data points with length m have distance <r then data points with length m+1 also have distance<r.

Sample entropy is defined as:

$$\mathrm{SampEn}(m, r, N) = -\log\left(\frac{A(r)}{B(r)}\right) \qquad [nu]$$

Where, A is the total number of template matches in m dimension; B is the total number of template matches in m+1 dimension. For example, it is known that aging reduces entropy and impaired systems exhibit lower entropy than healthy systems.

Gait Quantity Measures

Step Count (day and night included)

Sum of steps per calendar date (only during wear time) and averaged per week, calculated from walking bouts>= 3 seconds.

Number of Gait Bouts

Gait bouts are grouped and counted depending on their duration (5s ≤ x≤ 10s, 10s < x ≤ 20s, 20s < x ≤ 30s, 30s < x ≤ 60s, 60s < x ≤ 120 s, x > 120 s).

Signal Vector Magnitude (SVM) [mg]

Signal vector magnitude of the measured acceleration signal from three axes calculated for each 15 seconds epoch. This reflects physical activity.

The steps of the SVM algorithm:

1. Calculate SVM signal as
   SVM=sqrt(vAcc.^ 2 + mlAcc.^ 2 + apAcc.^ 2);
2. Filter the SVM signal fourth-order Butterworth band-pass filter between 0.5Hz and 20Hz.
3. The SVM signal is split into 15-sec windows (no overlap).
4. Per each day/night the mean and the sum of all windows are calculated.
5. The summed value is then normalized by the wear time in hours (per each day/night) to avoid bias in days/nights with low wear time.
6. The median value of all days/nights is extracted for both the mean and sum values.

Gait Detection

Gait events were estimated from a continuous wavelet transform (CWT, using the cwt MATLAB function) of the vertical acceleration signal which was first filtered, using 4th order Butterworth filter between 0.15 and 4 Hz. The wavelet transform decomposes signals over scaled and translated versions of the mother wavelet function, in our case Morlet wavelet. After the wavelet transform, all coefficients were summed and the envelope was calculated. A threshold on the summed coefficient signal was then applied to discriminate between gait events to non-gait events.

RESULTS

Table S1a: Comparing daily-living gait *quantity* measures pre intervention vs. immediate post intervention within 1 week

	**Pre intervention**	**Post intervention**	**P-value**
**Gait Quantity**			
Steps per day (average)	4496.50 [2513.00-7501.25]	4502.5 [2456.7-7384.0]	0.429
Mean SVM (g)	1.08 (±0.38)	1.13 (±0.41)	0.143
Sum SVM (g)	92430.83 (±31006.95)	91220.7 (±32549.8)	0.651
Percent Active (%)	8.08 (±2.20)	8.08 (±2.68)	0.992
Number of 5-10 seconds bouts/day	84.13 (±45.69)	87.55 (±54.40)	0.419
Number of 60-120 seconds bouts/day	2.5 [1.00-5.25]	2.75 [1.00-4.00]	0.625
Number of long bouts/ day (>120 sec)	2.00 [0.00-3.13]	1.00 [0.00-3.50]	0.277

Gait Quantity: Data are presented based on walking bouts equal to or longer than 6 seconds. Values are presented as mean+/-SD for continuous variables with normal distribution and median [IQR] for variables with abnormal distribution. Normal distribution is based on Kolmogorov-Smirnov test. P-Value is based on dependent t-test/Wilcoxon, P-Value<0.05

Table S1b: Comparing daily-living gait *quality* measures pre intervention vs. immediate post intervention within 1 week

		Pre intervention	Post intervention	P-value
Gait Quality				
Rhythm	Cadence (step/min)	102.85 [97.68-109.48]	102.02 [97.30-110.43]	0.413
	Stride Time (sec)	1.16 (±0.11)	1.17 (±0.13)	0.359
	Gait Speed (cm/sec)	89.00 (±18.49)	87.70 (±19.16)	0.389
Magnitude	frqV (Hz)	1.82 (±0.21)	1.82 (±0.24)	0.783
Consistency /regularity	Step Regularity V (nu)	0.49 (±0.16)	0.48 (±0.16)	0.269
	Stride Regularity V (nu)	0.44 (±0.14)	0.42 (±0.15)	0.210
	Step Asymmetry V (nu)	0.20 (±0.97)	0.21 (±0.11)	0.618
	Step Length (cm)	51.77 (±9.17)	51.26 (±9.20)	0.402
	Sample Entropy V (nu)	0.19 [0.14-0.22]	0.18 [0.14-0.24]	0.627
	Sample Entropy ML (nu)	0.21 (±0.80)	0.20 (±0.76)	0.386
	Sample Entropy AP (nu)	0.17 [0.13-0.21]	0.16 [0.12-0.20]	0.243

Gait Quality: Data are presented based on walking bouts equal or larger than 30 seconds, after removing irregular bouts. Values are presented as mean+/-SD for continuous variables with normal distribution and median [IQR] for variables with abnormal distribution. Normal distribution is based on Kolmogorov-Smirnov test. P-Value is based on dependent t-test/Wilcoxon, P-Value<0.05
